# The role of oxidative stress in neurodegenerative diseases and potential antioxidant therapies

**DOI:** 10.1515/almed-2022-0111

**Published:** 2022-12-19

**Authors:** Paula Sienes Bailo, Elena Llorente Martín, Pilar Calmarza, Silvia Montolio Breva, Adrián Bravo Gómez, Adela Pozo Giráldez, Joan J. Sánchez-Pascuala Callau, Juana M. Vaquer Santamaría, Anita Dayaldasani Khialani, Concepción Cerdá Micó, Jordi Camps Andreu, Guillermo Sáez Tormo, Isabel Fort Gallifa

**Affiliations:** Servicio de Bioquímica Clínica, Hospital Universitario Miguel Servet, Zaragoza, Spain; Instituto de Investigación Sanitaria Aragón (IIS Aragón), Zaragoza, Spain; Sociedad Española de Medicina de Laboratorio (SEQC-ML), Comisión de Estrés Oxidativo, Barcelona, Spain; Hospital General Universitario Gregorio Marañón, Madrid, Spain; Centro de Investigación en Red en Enfermedades Cardiovasculares (CIBERCV), Quebec, Spain; Universidad de Zaragoza, Zaragoza, Spain; Comisión de Lipoproteínas y Enfermedades Cardiovasculares, SEQC-ML, Barcelona, Spain; Hospital Universitari de Tarragona Joan XXIII, Tarragona, Spain; Comisión de Elementos traza, SEQC-ML, Barcelona, Spain; Servicio de Bioquímica Clínica y Patología Molecular, Hospital Clínico Universitario de Valencia, Valencia, Spain; Hospital Verge de la Cinta, Tortosa, Spain; UGD de Laboratorio, Hospital Regional Universitario de Málaga, Málaga, Spain; Dirección Médica Asistencial, Consorcio Hospital General Universitario de Valencia, Valencia, Spain; Universitat Rovira i Virgili, Tarragona, Spain; Hospital Universitari Sant Joan de Reus, Tarragona, Spain; Institut d’Investigació Sanitària Pere Virgili (IISPV), Tarragona, Spain; Centre Recerca Biomèdica, Tarragona, Spain; Unidad de Patología Oxidativa-UPOX-UV, Universidad de Valencia, Valencia, Spain; Servicio de Análisis Clínicos, Hospital Universitario Doctor Peset, Valencia, Spain; Laboratori ICS de Tarragona i Terres de l’Ebre, Tarragona, Spain

**Keywords:** antioxidants, biomarkers, neurodegenerative diseases, oxidative stress, reactive species

## Abstract

**Objectives:**

The central nervous system (CNS) is essential for homeostasis and controls the physiological functions of the body. However, the biochemical characteristics of the CNS make it especially vulnerable to oxidative damage (OS). This phenomenon compromises correct CNS functioning, leading to neurodegeneration and neuronal death.

**Contents:**

OS plays a crucial role in the physiopathology of neurodegenerative diseases. It is involved in multiple mechanisms of nucleic acid, protein, and lipid oxidation, thereby contributing to progressive brain damage. These mechanisms include mitochondrial dysfunction; excessive production of reactive oxygen and nitrogen species; deficiency of antioxidant defenses; protein oligomerization; cytokine production and inflammatory response; blood–brain barrier abnormalities; and proteasome dysfunction. All these dysfunctions are involved in the pathogenesis of neurodegenerative diseases, including Parkinson’s disease, Alzheimer’s disease, Huntington’s disease, or amyotrophic lateral sclerosis.

**Summary and outlook:**

A curative treatment is currently not available. Research is focused on the search for therapies that reduce oxidative damage and delay disease progression. In the recent years, researchers have focused their attention on the effects of antioxidant therapies.

## Oxidative stress and neurodegenerative diseases

**Figure 1:**
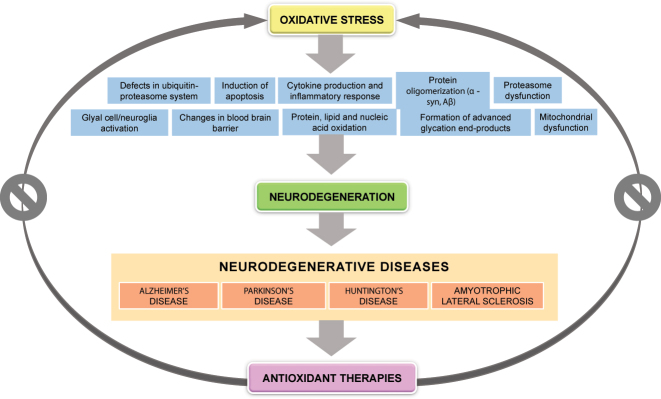
Oxidative stress and neurodegeneration. Oxidative stress may be involved in neurodegeneration via numerous etiopathogenic mechanisms, in neurodegenerative diseases like Alzheimer’s disease, Parkinson’s disease, Huntington’s disease, and amyotrophic lateral sclerosis. A range of antioxidant therapies have been tested in the recent years to reduce oxidative damage in neurodegenerative diseases.

Neurodegeneration is the process that involves progressive neuronal degeneration and death. This process compromises the correct functioning of the central nervous system [1]. Neurodegeneration is characteristic of a heterogeneous group of diseases known as “neurodegenerative diseases” (NDDs). In NDDs, oxidative stress (OS) occurs as a result of an imbalance between the production of oxidative species and the capacity of endogenous antioxidant defenses to neutralize or repair OS. Oxidative stress is involved in a variety of mechanisms, including nucleic acid, protein and lipid oxidation; formation of advanced glycation end-products; mitochondrial dysfunction; glial cell activation; apoptosis; appearance of defects in the ubiquitin-proteasome system; oligomerization of proteins such as alpha-synuclein (α-Syn) or beta-amyloid (Aβ); cytokine production and inflammatory response; blood–brain barrier (BBB) disruption; and proteasome dysfunction [2], [3], [4] (Figure 1).

The reactive oxygen species (ROS) involved in neurodegeneration include H_2_O_2_, the superoxide radical (O_2_^-^), and the hydroxyl radical (OH). The joint action of these species and reactive nitrogen species (RNS), including the nitric oxide radical (NO) and peroxynitrite (ONOO¯), has deleterious effects on neurons. The three major antioxidant enzymes are superoxide dismutase (SOD), catalase, and glutathione peroxidase (GPx). Of note, the redox potential of transition metals confers them crucial catalytic and structural roles in a variety of enzymatic processes. However, their capacity to accept or donate electrons makes them a potential source of free radicals and ROS. In this context, Fenton and Haber–Weiss reactions gain special relevance, where the hydroxyl radicals OH are derived from iron and H_2_O_2_ reduction [5].

The particularities of the brain make it especially vulnerable to oxidative damage, namely: (1) high energy consumption and metabolic demand, by which minimal imbalances cause tissue lesions and activate neuroinflammation mechanisms; (2) high oxygen consumption (20% of O_2_ entering the body), associated with high mitochondrial activity, and closely related to ROS production; (3) high lipid content of neuronal membranes, which are rich in polyunsaturated fatty acids and highly susceptible to ROS-induced lipid peroxidation, and metals with redox activity, including copper, iron, or zinc; and (4) neurons. Unlike other cells, neurons have weak antioxidant defenses and a low regenerative capacity, and their apoptotic mechanisms are strongly restricted to favor survival [3, 5].

Although low-moderate ROS and RNS concentrations play a major role in some physiological neuronal processes (i.e., signaling pathways, mitogenic response, anti-pathogen defense systems, and cell survival), overproduction and/or impaired activity of endogenous neutralizing mechanisms are involved in the pathogenesis of several NDDs. These diseases include Parkinson’s disease, Alzheimer’s disease, Huntington’s disease, amyotrophic lateral sclerosis, and others [6, 7]. In fact, there is evidence of changes in serum markers of OS in patients with degenerative diseases. This finding demonstrates the potential role of these molecules as potential diagnostic and prognostic biomarkers of NDD [2, 8, 9].

This review examined evidence available on the potential role of oxidative stress in the development of NDDs. Although the results of some studies are controversial, a description is provided of the advances made and studies carried out in the search for antioxidant therapies that reduce oxidative damage in NDDs.

### Alzheimer’s disease

Alzheimer’s disease (AD) is the most prevalent NDD in the elderly population. AD causes cognitive impairment and behavioral disorders, which typically manifest as loss of immediate memory and decline in other mental capacities. In neuropathological terms, AD is characterized by the accumulation of the β-amyloid peptide (Aβ) and tau protein (τ); extracellular amyloid plaques; intracellular accumulation of neurofibrillary tangles of hyperphosphorylated tau (τ); and loss of synaptic connections [7].

A range of studies suggest a critical role of ROX and OS in the pathogenesis of AD explained by their deleterious effects on molecules, especially on proteins. Recent analyses of the post-morten AD brain revealed the presence of oxidative damage to key enzymes involved in energy metabolism, proteins involved in neurotransmitter-related proteins, mitochondrial proteins, and proteasomal components [10]. Whether the increased formation of ROS is a primary cause or an effect of AD is still a matter of debate; however, several studies demonstrated severe OS in early stages of AD, even before significant Aβ accumulation occurs [11].

There is an association between oxidative imbalance induced by amyloid plaques and elevated levels of lipid peroxidation (i.e., malondialdehyde [MDA], 4-hydroxynonenal [HNE]); protein oxidation (i.e., carbonyl); nucleic acid oxidation (i.e., 8-hydroxydeoxyguanosine [8OHdG], 8-hydroxylguanosine); and reduced levels of antioxidants (uric acid, albumin, bilirubin, lycopene, vitamins A, C, and E, and enzymes such as SOD and catalase) in AD patients [6, 9]. OS may also promote the production and aggregation of Aβ and facilitate τ phosphorylation, thus forming a vicious cycle that promotes the pathogenesis of AD [12]. Along with ROS elevation, an increase in RNS has been described in AD patients (i.e., ONOO¯), which may cause similar changes in biomolecules and contribute to Aβ accumulation and τ phosphorylation [11, 13, 14]. Likewise, increased nitric oxide synthase (NOS) expression is directly related to Aβ deposits. This finding suggests that Aβ aggregates may cause NOS to produce nitric oxide, thereby resulting in the formation of 3-nitrotyrosine (3-NT) which favors the development of OS [15].

There is cumulative evidence that an abnormal distribution of metals such as Zn^2+^, Fe^3+^, and Cu^2+^ may contribute to Aβ aggregation and Aβ-induced oxidative stress and, potentially, to tau pathology [16]. Finally, elevated levels of ROS stimulate the transcription of pro-inflammatory genes and the release of chemokines and cytokines such as IL-1, IL-6, and TNFα, leading to a chronic neuroinflammatory and OS status that ultimately results in the loss of neurons and immune sensitivity [7, 10].

As described in this review, disturbed Cu, Zn, and Fe distribution is more deleterious than the accumulation of bulk tissue and may contribute to Aβ-induced OS and, potentially, to Tau disease.

Our research group and other groups have provided evidence in the last two decades that biometals (Cu, Zn, and Fe) are not only involved in Ab aggregation, but also facilitate ROS generation mediated by Ab:Cu2+ ligands.

### Parkinson’s disease

Parkinson’s disease (PD) is the second most common NDD in the population older than 60 years. PD is characterized by motor symptoms such as resting tremor and rigidity. As PD progresses, the dopaminergic neurons of the substantia nigra deteriorate and insoluble aggregations of α-Syn, known as Lewy bodies, accumulate [15].

Although the pathways and mechanisms of PD are still unclear, the finding of elevated levels of lipids, proteins, and oxidized nucleic acids, along with reduced levels of endogenous antioxidant systems such as SOD, catalase, reduced glutathione (GSH), and glutathione peroxidase in the substantia nigra of PD patients suggests that ROS, RNS, and OS play a role in the process [5, 17]. Specifically, an early biochemical change observed in PD is the selective loss of GSH in the substantia nigra, but not in other regions of the brain [6]. Elevated concentrations of MDA and HNE have also been reported in other body fluids, including plasma or cerebrospinal fluid (CSF) [18]. Likewise, higher levels of other oligoelements (Fe^3+^, Mn^2+^, Zn^2+^, Se^2+^, Cu^2+^, and Al^3+^) may be involved in the neurodegenerative process and exacerbation of cell damage that occur in the substantia nigra as a result of lipid peroxidation [19].

Some authors suggest that OS mediates posttranslational modifications in α-Syn, which include oxidation, nitration, and formation of HNE covalent adducts, thereby promoting α-Syn oligomerization [10, 20]. Finally, recent studies confirm that neurons with mutations in PD-related proteins, such as α-Syn, DJ-1, LRRK2, PINK1, and parkin, may be more vulnerable to increased OS and mitochondrial dysfunction [11, 21].

### Huntington’s disease

Huntington’s disease (HD) is a NDD characterized by motor dysfunction, dementia, and progressive cognitive decline. HD is caused by the abnormal expansion of CAG triplets encoding the aminoacid glutamine, located in exon 1 of the huntingtin gene (*HTT*). Although HD is mainly induced by mutated HTT protein toxicity (mHTT), other factors and processes associated with neuronal death may also be involved. Some of these factors include incorrect protein folding, abnormal proteolysis, protein aggregation, excitotoxicity, and OS, to name a few [8, 20, 22].

OS plays a key role in the course of HD; therefore, it can be used as a marker of disease course, prognosis and response to treatment. In HD, oxidative imbalance occurs before the onset of symptoms, which suggests that OS and reactive oxygen species production play a crucial role in neurodegeneration [23, 24]. Oxidative damage in these patients may be caused by the presence of mHTT deposits resulting from increased ROS levels. In addition, there is evidence that DNA repair induced by oxidative damage could lead to the expansion and instability of CAG repeats in the mutated *HTT* gene, thereby promoting aggregation and inducing neuronal death [8, 20, 25].

Several biomolecules have been suggested as potential biomarkers of OS. Firstly, OS increases damage to DNA (8OHdG); proteins (carbonyl groups and protein nitration, 3-NT); and lipids (MDA, HNE, and thiobarbituric acid reactive substances), among other [22, 26]. In addition, there is a decrease in GSH content and an increase in levels of antioxidant enzymes such as GPx, catalase, and SOD [23].

Mitochondrial dysfunction is considered a key defect in the pathogenesis of HD, since it causes and increase in ROS, oxidative damage, and neuronal death. OS also contributes to the excitotoxicity of glutamate, the most abundant excitatory neurotransmitter in the central nervous system. Free radicals inhibit glutamate uptake by glyal cells, thereby increasing its concentrations. Excitotoxicity is also associated with excessive influx of calcium ions into the cell. The resulting calcium overload leads to the activation of the neuronal NOS and subsequent release of NO·, which will convert into ONOO¯ after reacting with O_2_^-^ from the electron transport chain. This reaction induces an imbalance between oxidant and antioxidant systems. This imbalance is characterized by excessive production and release of ROS and RNS and a decline in anti-oxidant defense systems [23].

### Amyotrophic lateral sclerosis

Amyotrophic lateral sclerosis (ALS) is characterized by the progressive degeneration of upper and lower motor neurons in the spinal cord, the cerebral cortex, and the brain stem. OS induced by the production and accumulation of ROS is a contributing factor to ALS due to the abnormal energy metabolism it causes. It is related to age, environmental factors, or excitotoxicity mediated by glutamate, resulting in excessive production of free radicals [6].

Some mechanisms involved in OS and damage to motor neurons have been identified. Around 90% of ALS cases are sporadic, but the remaining 10% result from mutations in the *SOD1* gene. This gene encodes SOD, the most important neuronal antioxidant enzyme. SOD catalyses the scavenging of superoxide radicals in a dismutation reaction, which results in an increase in ROS and oxidative damage. A number of OS biomarkers have been identified in proteins (carbonylated proteins, 3-NT); lipid peroxidation products; and in DNA and RNA oxidation products (8OHdG) in the blood, urine, CSF, and tissue of ALS patients. These findings demonstrate that OS and ROS play a key role in brain damage [27, 28]. Mutations in the *SOD1* gene also induce mitochondrial dysfunction. This is central to disease associated with defective oxidative phosphorylation, ROS production, and abnormal calcium retention capacity, which may promote OS [28], [29], [30].

## Anti-oxidant therapies

Given the pivotal role that oxidative stress plays in neurodegeneration, modulating free radical production or alleviating their harmful effects have been suggested as potential therapeutic strategies for the prevention and control of NDDs [2]. Different antioxidant compounds have been investigated for their neuroprotective effects, as a strategy to reduce OS-induced damage in NDDs [15]. The most relevant limitation of this strategy is ineffectiveness in the administration of medicines, due to the limited BBB permeability. This drawback prevents these molecules from being incorporated to NDD therapeutics [3, 5, 21].

### Alzheimer’s disease

The evidence provided by the majority of the studies on the effects of antioxidant substances (vitamin E, vitamin C, lipoic acid, coenzyme Q10, and derivatives) is not conclusive. Thus, studies demonstrate a reduction of OS, but failed to demonstrate any beneficial effects on cognitive or functional function [6, 21].

Antioxidants like coenzyme Q10 or its derivative mitokine mesylate have been proven to substantially reduce amyloid plaques in transgenic mouse models of AD [31, 32]. Despite the encouraging results obtained, escalation to clinical trials in human beings has not been reported. Other treatments like latrepirdine or acetyl-L-carnitine (ALCAR) have been tested in clinical trials. However, none has conclusively proven these treatments to be effective. The reason may be that apparent elimination of oxidative stress may not reverse existing changes, such as Aβ accumulation or τ hyperphosphorylation [13]. Omega-3 fatty acid supplementation in mild AD has demonstrated to confer beneficial effects in patients with slight impairment of brain function. However, although some studies report changes in scales of cognitive function in more severe cases, they are not enough to support omega-3 fatty acid supplementation in the treatment of AD [33].

The most promising results were obtained in a study with resveratrol. This compound minimizes Aβ peptide aggregation and promotes survival and tolerance to OS in the central nervous system by exerting neuroprotective effects [15, 34].

Monoamine aoxidase inhibitors (MAOIs) are currently used in the treatment of neurodegenerative disorders such as AD. Thus, MAOIs prevent tau hyperphosphorylation, OS, and Aβ accumulation by exerting neuroprotective effects. The most frequently tested MAOIs are selegiline (IMAO-B) and ladostigil (IMAO-A and B). The evolution of these compounds combined with iron-chelating activity could be effective therapeutic targets in the treatment of NDDs like AD [35] (Table 1).

**Table 1:** Clinical trials with agents/drugs with antioxidant properties in neurodegenerative diseases.

Antioxidant therapy	Family	Disease	Mechanism of action	Results	Reference
Resveratrol	Polifenol	AD	It reduces MDA and nitrite production, restores GSH levels and reduces SOD activity.	Studies *in vivo* e *in vitro* demonstrate that resveratol may be useful for the treatment of AD not only for its antioxidant effects, but also for its neuroprotective and antiinflammatory effects.	Islam et al. [34]
Lipoic acid	Thiol	AD	It upregulates levels of the enzyme glutathione reductase and reduces MDA by increasing antioxidant activity.	It is a supplement with a significant potential in the treatment of AD, and a candidate with positive effects on mitochondrial dysfunction.	Kaur et al. [36]
Acetyl-L-carnitine	Acetylated form of L-carnitine	AD	It attenuates cytotoxicity, protein oxidation and lipid peroxidation significantly. It increases levels of cellular GSH.	It helps patients maintain their initial cognitive performance and attenuates the behavioral and psychological symptoms of dementia during treatment.	Mota et al. [37]
Omega-3 acid	Polyunsaturated fatty acid	AD	It reduces lipid peroxidation and increases SOD, catalase and GPx activity.	It exerts beneficial effect in the onset of disease in the presence of mild brain function impairment.	Canhada et al. [33]
Rasagilina, selegilina	IMAO-B	AD	It increases antioxidant and airon chelator activity.	It prevents tau protein hyperphosphorylation and oxidative stress. It regulates Aβ deposits. It may improve memory and learning capacities.	Behl et al. [35]
N-acetylcysteine	Thiol	PD	It reduces lipid peroxidation and SOD activity. It increases GPx and GSH activity.	It seems to induce significant changes in GSH concentrations in CSF, albeit without an immediate improvement of symptoms.	Niedzielska et al. [6]
Zonisamide	Sulfonamide	PD	It inhibits glutamate release. It increases levels of 8-OHdG in urine of PD patients.	It is effective in improving motor symptoms as adjuvant agent of standard therapy.	Niedzielska et al. [6]
Deferiprone	Iron chelator	PD	It has capacity to rescue iron-overloaded cells, especially mitochondria (the organelles most affected by cell iron accumulation), and reduce ROS formation and the resulting oxidative stress.	The patients who received deferiprone exhibited a significantly better motor performance at 6 or 12 months.	Devos et al. [38]
Safranal	Apocarotenoid	HD	It prevents the elevation of 3-NT and MDA levels. It reduces SOD, catalase and GSH activity.	It exerts beneficial effects on motor activity and oxidative brain damage in an animal model.	Fotoohi et al. [39]
Epigallocatechin gallate	Polyphenol	HD	It reduces reactive oxygen species and chelation of bonding metal ions.	It helps reduce mHTT accumulation.	Sebastiani et al. [40]
Riluzol	Benzothiazole	ALS	It inhibits excessive glutamate production by reducing levels of cytosolic calcium.	It exerts slight beneficial effects by delaying disease progression and increasing survival.	Orrell et al. [41]
			It induces glutathione synthesis thereby reducing ROS.		Jaiswal et al. [30]
Edaravone	Pirazoline	ALS	It eliminates lipid peroxides and hydroxyl radicals. It reduces levels of 3-NT.	It delays progression of functional motor disorders by reducing oxidative stress in ALS patients.	Yoshino et al. [42]
					Jaiswal et al. [30]
Curcumin	Phenolic compound (diferuloylmethane)	ALS	It attenuates oxidative damage and mitochondrial dysfunction.	It slightly slows down disease progression by modulating mitochondrial functions, thereby improving redox state and improving oxidative damage.	Chico et al. [43]
Vitamin E	Tocopherol	PD	It attenuates the effects of ROS and inhibits lipid peroxidation.	It was not found to delay functional decline or improve the clinical symptoms of PD.	Shoulson et al. [44]
Coenzyme Q10 and derivatives (mitokine mesylate)	Ubiquinone	AD	It reduces levels of MDA and lipid peroxidation. It increases SOD and mitochondrial complex I activity. It reduces oxidative damage.	It improves cognitive decline, oxidative stress, synaptic loss and reduces Aβ accumulation in transgenic animal models of AD.	McManus et al. [32]
		PD		It plays a limited role in the treatment of PD, since it does not delay functional decline or improve symptoms in PD patients.	Zhu et al. [45]
		HD		The trial does not provide evidence that coenzyme Q10 delays functional decline in HD, and the results do not provide a rationale for this compound to be recommended as a treatment for HD.	McGarry et al. [46]

3-NT, 3-nitrotyrosine; 8-OHdG, 8-hydroxydeoxyguanosine; Aβ, beta-amyloid protein; AD, Alzheimer’s disease; HD, Huntington’s disease; ALS, amyotrophic lateral sclerosis; PD, Parkinson’s disease; GPx, glutathione peroxidase; GSH, reduced glutathione; B-MAOI, monoamine oxidase B inhibitors; CSF, cerebrospinal fluid; MDA, malondialdehyde; mHTT, mutated HTT protein; ROS, reactive oxygen species; SOD, superoxide dismutase.

These results suggest a central role of OS in AD. Antioxidant therapies induce a reduction of OS products. The low efficacy or failure of clinical trials may be explained by the fact that the pathological changes occurred in AD, such as Aβ accumulation and τ hyperphosphorylation are not effectively modified, even when OS is reduced [13].

### Parkinson’s disease

In the light of the growing evidence on the role of OS in Parkinson’s disease, different studies have been conducted in an attempt to reduce OS in PD patients. Unfortunately, recent reviews and meta-analyses reveal that supplementation with the Q10 coenzyme, an essential cofactor involved in mitochondrial oxidative phosphorylation–and, therefore, a potent antioxidant–, plays a limited role in the treatment of PD. The reason is that this therapy does not delay functional decline or confers any symptomatic benefit to PD patients [31, 45]. Likewise, N-acetylcysteine seems to induce significant changes in GSH concentrations in CSF, albeit without an immediate improvement of symptoms [6] (Table 1).

Deferiprone, an iron chelator, has been tested to reduce the increase in iron concentrations in the substantia nigra of PD patients and prevent ROS production. This agent has been proven to eliminate iron deposits and increase GPx and SOD activity in CSF, thereby reducing ROS formation and the resulting oxidative stress [6, 38]. Vitamin E has also been suggested as a strategy to diminish OS, since it attenuates ROS effects and inhibits lipid peroxidation. This compound alleviates clinical symptoms, but no evidence has been provided of a beneficial effect on functional decline [6, 44].

Zonisamide, an anticonvulsant agent, has been used to treat resting tremor in AD, since it inhibits the increase in levels of 8-OHdG in urine. Hence, the use of zonisamide could be a useful strategy against oxidative damage [6] (Table 1).

The application of antioxidant therapies is grounded on the reduction of ROS and RNS observed in PD patients. Although the studies conducted in animal models demonstrate their effectiveness, structural differences with the human brain limit their application in human beings. Therefore, the results of these studies should be interpreted with caution [47].

### Huntington’s disease

There is no current cure for HD, and the therapies used are aimed at reducing chorea and mental disorders. A variety of antioxidant therapies have been investigated to reduce oxidative damage and delay disease progression.

A study revealed that antioxidant treatment with coenzyme Q10 was not effective in animal models of HD. Thus, Q10 did not prevent deterioration of mitochondrial respiratory chain function or improve survival or mortality rates. This compound alone is not enough to delay or halt HD progression [31].

A delay demonstrated that safranal, an organic compound with antioxidant activity isolated from saffron, prevents motor dysfunction in animal models of HD. This effect is explained by the potential preventive effect of this compound on the elevation of nitrite and MDA levels, and the reduction of SOD, catalase activity, and GSH [39].

Epigallocatechin gallato (EGCG), the most abundant catechin in tea, is a polyphenol used in a wide variety of dietary supplements. The beneficial effects of this compound include the elimination of free radicals, the reduction of reactive oxidative species or chelation of bonding metal ions. This activity may reduce mHTT accumulation and attenuate toxicity in models of HD *in vivo* [40] (Table 1).

### Amyotrophic lateral sclerosis

The hypothetic role of OS in ALS has been investigated in a variety of experimental trials. Currently, there is no cure for this disease, and the only treatment approved by the FDA is riluzol, an antagonist of glutamate. This agent blocks excessive glutamate release, thereby reducing cytosolic calcium levels, with collateral effects on ROS production and mitochondrial function. There is evidence demonstrating that this agent reduces ROS by inducing glutathione synthesis [28], [29], [30]. Likewise, riluzol exerts slight beneficial effects by delaying disease progression, thereby increasing survival [28, 41].

Several pharmacotherapeutic agents with antioxidant properties have been used to delay ALS progression. A recent study demonstrated that antioxidants like edaravone (approved by the FDA in 2017) are effective in delaying disease progression at early stages and in slowing functional decline in some patients [15, 28, 42]. This agent eliminates oxygen radicals, including nitric oxide radical and peroxynitrite anion [30].

A study on curcumin therapy in ALS patients provided encouraging results that showed a slight delay in disease progression. Curcumin modulates mitochondrial functions, thereby improving redox state and reducing the oxidative damage caused [28, 43] (Table 1).

## Conclusions

Research on the role of oxidative stress in NDDs has provided valuable information on the underlying pathogenic mechanism of these diseases. Thus, studies have improved our understanding of these diseases and provided guidance on the search for diagnostic, prognostic, and follow-up biomarkers. Imbalance between reactive species production and antioxidant defense systems seems to play a central role in neurodegeneration. The CNS is very vulnerable to oxidative damage, which increases susceptibility to neurodegeneration and results in neuronal death and progressive brain damage. In the light that there are no curative treatments available for these diseases, the use of antioxidant agents emerges as a promising approach to tackle neurodegeneration.

Notably, antioxidant therapies reduce OS by acting indirectly on the mechanisms that cause imbalance. The mechanisms involved in the genesis of ROS and RNS should be further investigated to find the way to inhibit ROS and RNS production and prevent imbalance before it causes metabolic changes. Although most antioxidant therapies are based on the assumption that oxidative damage contributes to neurodegeneration in NDDs, research on this subject is limited. Therefore, further studies are needed to improve knowledge on the role of OS in the physiopathology of NDDs and its potential use in clinical practice. In addition, the knowledge generated may guide the development of effective treatments.

The findings of this review highlight the need for further clinical trials that explain the role of the mechanisms involved in the genesis of ROS and RNS in NDDs. New therapies should be aimed at delaying ROS and RNS production and prevent oxidative imbalance before it causes metabolic changes. The treatments currently available still have a limited efficacy. The identification of the metabolic pathways involved in OS would pave the way to new therapies targeted to the origin of OS rather than its products.
